# Household Effects of School Closure during Pandemic (H1N1) 2009, Pennsylvania, USA

**DOI:** 10.3201/eid1608.091827

**Published:** 2010-08

**Authors:** Thomas L. Gift, Rakhee S. Palekar, Samir V. Sodha, Charlotte K. Kent, Ryan P. Fagan, W. Roodly Archer, Paul J. Edelson, Tiffany Marchbanks, Achuyt Bhattarai, David Swerdlow, Stephen Ostroff, Martin I. Meltzer, for the Pennsylvania H1N1 Working Group

**Affiliations:** Centers for Disease Control and Prevention, Atlanta, Georgia, USA (T.L. Gift, R.S. Palekar, S.V. Sodha, C.K. Kent, R.P. Fagan, W.R. Archer, P.J. Edelson, A. Bhattarai, D. Swerdlow, M.I. Meltzer); Pennsylvania Department of Health, Harrisburg, Pennsylvania, USA (T. Marchbanks, S. Ostroff)

**Keywords:** Influenza A virus, influenza, H1N1 subtype, pandemic (H1N1) 2009, compliance, economics, school closure, Pennsylvania, viruses, dispatch

## Abstract

To determine the effects of school closure, we surveyed 214 households after a 1-week elementary school closure because of pandemic (H1N1) 2009. Students spent 77% of the closure days at home, 69% of students visited at least 1 other location, and 79% of households reported that adults missed no days of work to watch children.

Some studies have suggested that school-age children are influential in the ongoing transmission of influenza (*1**,**2*). Closing schools may potentially reduce the spread of influenza (*3**,**4*). In mid-May 2009, an elementary school (kindergarten-4th grade) in a semirural area of Pennsylvania closed for 1 week after an abrupt increase in absenteeism due to influenza-like illness (ILI) and the confirmation of influenza A pandemic (H1N1) 2009 virus infection in 1 student. Other schools in the district remained open. From May 26 through June 2, 2009, investigators from the Pennsylvania Department of Health and the Centers for Disease Control and Prevention surveyed households with students at the school by telephone to assess influenza symptoms, childcare arrangements, movements of affected children during the school closure period, and household demographics and socioeconomic status. This study did not address the transmission effects, but assessed the potential disruption to households resulting from school closure.

## The Investigation

The survey was considered a public health response. School administrators provided contact information for households with children attending the school. Investigators asked to speak to an adult in the household. If an adult was available and consented, the survey was administered. For each day of school closure, respondents were asked for the following information: where the student spent most of the day; whether the student went elsewhere (prompted by specific venues), who watched the student; and whether the person watching the student missed work. Questions were asked regarding the oldest student if multiple children attended the school.

Respondents were also asked, for each household member, whether the person had symptoms of ILI (defined as fever with cough and/or sore throat) between May 1, 2009, and the time of the survey. Children were defined as persons <18 years of age, and those >18 years of age were considered adults. The Technical Appendix describes the process followed to calculate variables used in the analysis.

The locations where students spent most of the day and other venues visited were tabulated. Significant differences in venues visited by students with and without ILI were determined by using the Fisher exact test. We computed unadjusted and adjusted odds ratios (ORs) for the following characteristics versus whether the household reported missing >1 workdays: whether the oldest student reported ILI (repeated for whether any adult, any student at the closed school, or any child in the household reported ILI), whether the household had a single child, whether the household had just 1 adult, whether all adults in the household worked outside the home, and whether household income was above the median (Technical Appendix). Adjusted ORs were computed in a logistic regression model for variables that had unadjusted ORs significant at p<0.10 by the Fisher exact test.

Surveys were completed for 214 (59%) of 364 households (59%), and accounted for 269 (59%) of the 456 students enrolled at the school. Table 1 shows the demographics of surveyed households. Most households had at least 2 adults, at least 2 wage earners, and >2 children. Households with incomes >$60,000 were at or above the median income. Because some of the oldest students spent days in multiple locations during the 5 days of school closure, we calculated the number of student-days at each venue (number of students at each type of venue multiplied by the number of days spent there). Home was the primary location during the school closure for 77% of the student-days (Technical Appendix Figure 1). The next most common location was another family member’s home.

**Table 1:** Demographic variables of households affected by school closure during pandemic (H1N1) 2009, Pennsylvania, USA*

Variable	No. (%) households†
No. adults (>18 y)	
1	25 (11.7)
2	157 (73.4)
>2	32 (15.0)
No. children (<18 y)	
1	44 (20.6)
2	92 (43.0)
3	53 (24.8)
>3	25 (11.7)
Households with >1 adult with ILI	34 (15.9)
Households with >1 child with ILI	88 (41.1)
Households with the oldest student with ILI	67 (31.3)
Household income (US$)	
0–29,999	27 (12.6)
30,000-59,999	65 (30.4)
60,000–89,999	51 (23.8)
>90,000	42 (19.6)
Don’t know/refused/missing	29 (13.6)
No. wage earners	
1	64 (29.9)
2	135 (63.1)
>3	12 (5.6)
Don’t know/refused/missing	3(1.4)
Time adult in household missed work to watch oldest student, d	
0	168 (78.5)
1	13 (6.1)
2	7 (3.3)
3	4 (1.9)
4	4 (1.9)
5	18 (8.4)
% Adults in household who work	
33	5 (2.3)
40	1 (0.5)
50	44 (20.6)
67	16 (7.5)
75	3 (1.4)
100	142 (66.4)
Don’t know/refused/missing	3 (1.4)

*ILI, influenza-like illness. †Categories are mutually exclusive and exhaustive, but percentages may not sum to 100% due to rounding.

Sixty-nine percent of students visited other venues during school closure (Technical Appendix Figure 2). Those reported as having ILI were more likely to have visited a healthcare provider than those without ILI (p<0.01), but no other statistically significant differences were found in terms of venues visited between those with ILI and those without ILI. Seventy-nine percent of households reported zero missed workdays (Table 1); of the remaining households in which work was missed, ≈40% missed work during all 5 days of school closure.

The only household characteristics for which the OR for missing any workdays was significantly different from 1 at p<0.10 were single child, all adults work, and household income is greater than or equal to median income (Table 2). When adjusted ORs were calculated, household income greater than or equal to median was significant at p<0.05, but because income data were only available for 184 households (vs. 214 for the other factors), the sample on which the adjusted ORs were calculated was somewhat different. All adults in the household working was significantly associated with household income greater than or equal to the median (p<0.01).

**Table 2:** Predictors of households reporting days of work missed to watch children during school closure for pandemic (H1N1) 2009, Pennsylvania, USA*†

Variable	OR	Adjusted OR‡
Oldest student with ILI	1.22	
Any student with ILI	1.20	
Any child with ILI	1.16	
Any adult with ILI	1.67	
Single adult	1.50	
Single child	2.02§	2.02§
All adults work	2.35¶	2.08
Household income above median income	2.62¶	2.31¶

*OR, odds ratio; ILI, influenza-like illness. †When a household had >1 child attending the school that was closed, we asked about time taken from work to watch the oldest child. ‡Adjusted OR estimated by logistic regression. §p<0.10. ¶p<0.05.

## Conclusions

Estimating the economic effects of school closure can provide useful information to aid in estimating whether it is likely to achieve the intended goals. Households that reported missed work incurred costs, even if those costs were only in terms of lost vacation or sick time.

The data show that most of the oldest students spent the days of school closure at home. However, most students left the home at least once during the closure period to visit routine venues (stores, locations of sports events or practices, restaurants). Few differences were found for reported ILI (with the obvious exception that students with ILI had significantly more visits to healthcare providers). These latter 2 findings are similar to those found in a 2006 study of an influenza B–related school closure in North Carolina, USA (*5*). This behavior, particularly by students who reported ILI, may increase the risk for onward transmission. A survey of 2 school districts in Kentucky that experienced a seasonal influenza–related school closure also found that students engaged in many activities outside the home (*6*), as did a survey of households affected by pandemic (H1N1) 2009 school closure in Australia (*7*).

In our study, only 22% of households reported missing any work to watch the students, fewer than during the closure in Australia (*7*). However, in ≈40% of households in which work was missed, an adult missed work for all 5 days of closure, indicating a relatively large effect on those households (Table 1). A limitation is that the question regarding missed work was narrowly worded (Technical Appendix) and did not explore whether an adult missed work for other reasons. As shown in Table 2, adult ILI was not significantly associated with missing work. Some adults with ILI may have stayed at home to watch students but determined that they would have stayed home because of their own illness had the school not been closed and answered “no.” In the Kentucky school closure situation, 29% of households had working adults who provided childcare. In 16% of households, adults missed work and lost pay (*6*). Closures for >1 week may result in more households that report missing work days. The factors “all adults working” and “having a household income equal to or greater than the median” were associated with missed workdays, as were fewer children (other children in the home may have made it possible for some households to avoid having an adult miss work to watch students whose school was closed).

These findings add to the body of literature on the effects of school closure on households. They can be used by decision makers, as well as parents, to assess the potential social disruption of school closure in the context of future influenza outbreaks.

## Supplementary Material

Technical Appendix Derivation of Variables Used in Analysis.
