# Supplementary material for: Household Effects of School Closure during Pandemic (H1N1) 2009, Pennsylvania, USA
Source: Emerg Infect Dis. 2010 Aug;16(8):1315–7. doi: 10.3201/eid1608.091827 (PMC3298323; doi:10.3201/eid1608.091827)
Supplement: Technical Appendix — Derivation of Variables Used in Analysis. [file 09-1827-Techapp.pdf]

Article DOI: 10.3201/eid1608.091827

Household Effects of School Closure during Pandemic (H1N1) 2009, Pennsylvania, USA

Technical Appendix

Derivation of Variables Used in Analysis

Respondents were asked questions regarding the childcare arrangements and locations visited outside the home for each of the days the school was closed. To determine who watched the child, the question asked for each day was, “On [given day], who watched [name of oldest child at the school]?” To determine whether the person watching the child had to miss work, the respondents were asked, “Did the person watching [name of oldest child at the school] have to miss work?” To determine where the student spent most of the day, the question was, “On [given day], where did [name of oldest child at the school] spend most of the day?” (online Technical Appendix Figure 1). For the questions concerning child care arrangements, on the second through fifth day of school closure, respondents were asked, “Were the child care arrangements the same as yesterday?”

To assess what other venues the student may have visited, respondents were asked, “In addition to where [name of oldest child at the school] spent most of the day, did they go anyplace else?” Respondents were provided with the following list for each day: library, sports practice or game, mall or other shopping site, restaurant (except drive-through), drive-through restaurant, party, hang out with friends (other than a party), church function or services, movie, or other. For respondents who indicated an “other” venue, survey takers wrote in the answer provided, which was then entered in the database as a text field. When responses were aggregated, a large number of health care provider visits and visits to family members were noted; these were identified as additional venues (online Technical Appendix Figure 2). Also, some of the “other” venues included visits to concerts, which were aggregated with movies (online Technical Appendix Figure 2).

The daily data were summed for the variables on number of missed workdays and venues visited used in the analyses. For example, if the respondent indicated that an adult missed work to watch the oldest student on the first and third days of school closure, the number of missed workdays for that household would be 2 (Table 1). If, on any given day, the respondent indicated that, "the child care arrangements were the same as yesterday," the previous day was examined to see the details for the child care arrangements; this process continued until the details could be ascertained. The same process was followed to determine the number of days the oldest students spent at each location. The number of times during the period of school closure that each of the outside venues was visited was determined by adding the number of days for which a given venue was identified.

The percentage of adults in the household whose work was calculated by dividing the number of wage earners by the number of adults. This number was truncated at 100 (in some cases, persons in the household <18 years of age could have been wage earners, and the result could have led to a percentage >100%). Household median income was assessed by determining the median category for household income based on the responses to the household income question. Single adults and single children were determined by identifying households that only indicated 1 person ≥18 years of age or 1 person <18 years of age, respectively.

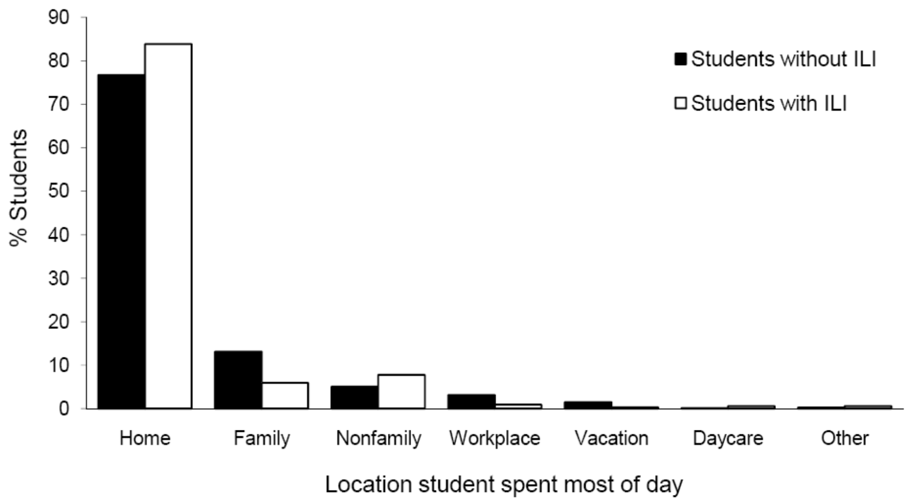

Technical Appendix Figure 1. Location where oldest students spent most of the day on days when school was closed for pandemic (H1N1) 2009, Pennsylvania, USA. ILI, influenza-like illness; family, someone else's home (family); nonfamily, someone else's home (nonfamily); workplace, parent/guardian workplace.

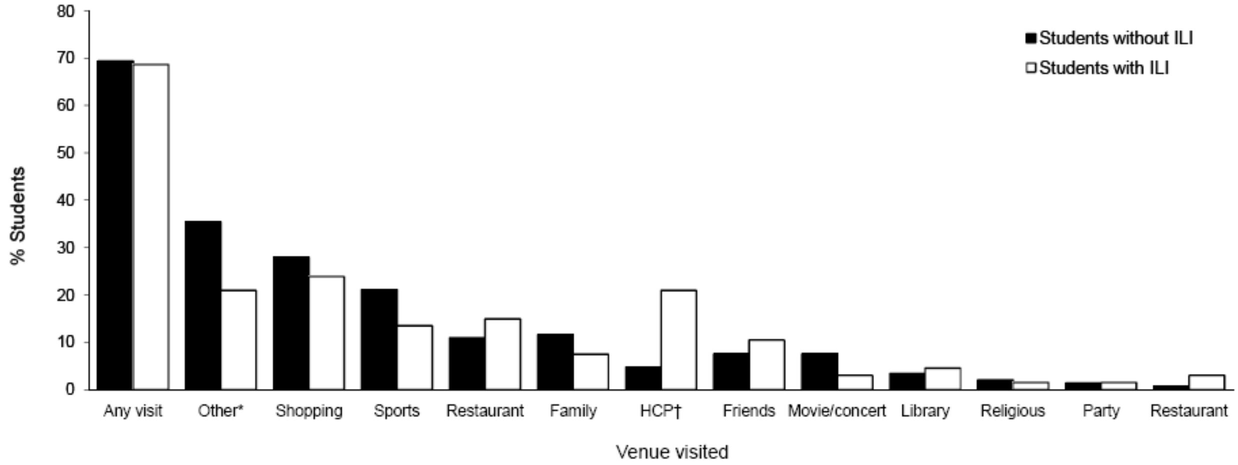

Technical Appendix Figure 2. Other venues visited by the oldest students on days when school was closed for pandemic (H1N1) 2009, Pennsylvania, USA. ILI, influenza-like illness; sports, sports practice or game; family, visiting family; HCP, visiting health care provider; friends, hanging out with friends; religious, religious function or service; party, attending a party; restaurant, drive-through restaurant. *p<0.05. †p<0.01.
